# P02.181. Heart rate variability and peripheral temperature during whole body immersion at different water temperatures

**DOI:** 10.1186/1472-6882-12-S1-P237

**Published:** 2012-06-12

**Authors:** F Edelhäuser, S Goebel, C Scheffer, D Cysarz

**Affiliations:** 1 University of Witten/Herdecke, Herdecke, Germany

## Purpose

Whole body immersion in water (WI) constitutes a significant role in the area of CAM as well as in rehabilitation facilities. WI has strong effects on the autonomic nervous system. In this study, we investigate the effects of different water temperatures (33°C, 36°C, and 39°C) on heart rate variability (HRV), peripheral and core body temperature (PT and CBT, respectively).

## Methods

Twenty-one healthy subjects (age: 24.3 ± 2.3 years, 11 female) underwent WI with water temperatures of 33°C (WI33), 36°C (WI36), 39°C (WI39). The procedure was: supine rest (30 minutes), WI (20 minutes) and supine rest (30 minutes). An ECG, the nasal/oral airflow, core body and temperature of extremities were recorded. The results of the last 5 minutes at the end of each interval are presented.

## Results

During WI33 and WI36 CBT decreased compared to rest before WI, whereas WI39 led to an increase of CBT. Peripheral temperature was determined by the water temperature. The average RR-interval increased during WI33 (970 ms) compared to rest before WI (910 ms), whereas it decreased during WI36 (850 ms) and WI39 (636 ms). The standard deviation of the RR-intervals (SDNN) was reduced during WI39 (26 ms), whereas it was augmented during WI33 (78 ms) compared to rest before WI (63 ms). The square root of the mean squared difference of successive RR-intervals (RMSSD) decreased during WI39 (14 ms) and WI36 (43 ms), whereas it increased during WI33 (72 ms) compared to rest before WI (52 ms).

## Conclusion

Each water temperature showed specific effects on CBT, PT and HRV during WI. WI39 lead to an increase of CBT and PT and a decrease of HRV whereas WI33 showed opposite effects. Hence, WI39 induces moderate cardiovascular stress and moderate hyperthermia whereas WI33 induces mild hypothermia and cardiovascular relaxation. The water temperature is crucial for therapeutic purposes.

